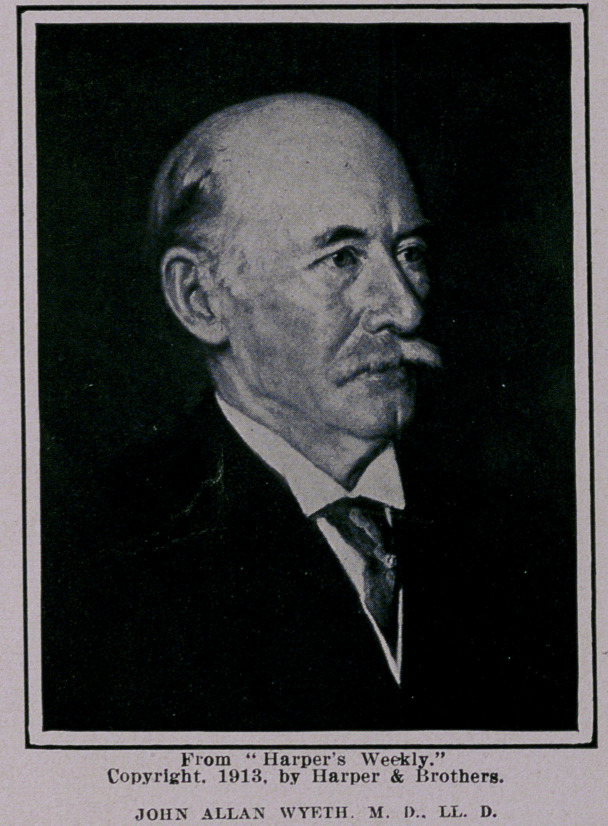# The South’s Great Surgeon

**Published:** 1913-08

**Authors:** 


### EDITORIAL DEPARTMENT.

DR. F. E. DANIEL, Editor DR. R. H. L. BIBB, Associate Edito r

DEPARTMENTS { EUGENICS: Drs. M. Duggan and T. Y. Hull.
WOMAN'S DEPARTMENT: Mrs. F. E. Daniel.

### The South's Great Surgeon.

An Example and an Inspiration.—We treat our readers to a picture of the great Wyeth, and publish herewith an extended account of the new Polyclinic, the creation of his brain and energy, a monument more lasting than brass or marble. The South is proud of the Polyclinic and proud of John A. Wyeth,—and with cause. Personally, he has been an ideal and has lived up to and realized it, and it should be known that the road that led to Fame's proud temple was no primrose path. Turned loose after the war, a youth without friends or means, he *qualified* in medicine, according to the very low standards of even the best colleges,—he realized that he was unfit to practice medicine. It was then the great concept of a post-graduate school was born in his brain, and he went into the swamps of Arkansas, not as a doctor, but as a laborer. He dedicated himself to a life of the hardest kind of labor. He built jails and courthouses by contract, undergoing almost untold hardships,—*but he succeeded.* He accumulated, in some two or three years, about $3500. With this sum he started to New York to found the first post-graduate medical college. Behold the result! And—we call him great, and he is great, and his life's struggles and triumphs will stand forever as an incentive to all young men in the pursuit of medicine to be not discouraged, but to press on to success. Some years ago the "Red Back" published an autobiography of Dr. Wyeth. It reads like a romance (especially a certain fish story), but it is true and not colored or exaggerated.

## Figures and Tables

**Figure f1:**